# Cell Propagation of Cholera Toxin CTA ADP-Ribosylating Factor by Exosome Mediated Transfer

**DOI:** 10.3390/ijms19051521

**Published:** 2018-05-19

**Authors:** Cristiana Zanetti, Angelo Gallina, Alessia Fabbri, Sofia Parisi, Angela Palermo, Katia Fecchi, Zaira Boussadia, Maria Carollo, Mario Falchi, Luca Pasquini, Maria Luisa Fiani, Massimo Sargiacomo

**Affiliations:** 1 Department of Oncology and Molecular Medicine, Istituto Superiore di Sanità, Viale Regina Elena 299, 00161 Rome, Italy; cristiana.zanetti@iss.it; 2 Department of Neurosciences, Istituto Superiore di Sanità, Viale Regina Elena 299, 00161 Rome, Italy; gallinaangelo@yahoo.com; 3 National Center for Global Health, Istituto Superiore di Sanità, Viale Regina Elena 299, 00161 Rome, Italy; alessia.fabbri@iss.it (A.F.); sofia.parisi91@gmail.com (S.P.); annica86@hotmail.it (A.P.); katia.fecchi@iss.it (K.F.); zaira.boussadia@iss.it (Z.B.); 4 Core Facilities–Cytometry Unit, Istituto Superiore di Sanità, Viale Regina Elena 299, 00161 Rome, Italy; maria.carollo@iss.it (M.C.); luca.pasquini@iss.it (L.P.); 5 National AIDS Center, Istituto Superiore di Sanità, Viale Regina Elena 299, 00161 Rome, Italy; mario.falchi@iss.it

**Keywords:** cholera toxin, exosomes, endocytic pathway, Caveolin-1, monosialganglioside GM1

## Abstract

In this study, we report how the cholera toxin (CT) A subunit (CTA), the enzyme moiety responsible for signaling alteration in host cells, enters the exosomal pathway, secretes extracellularly, transmits itself to a cell population. The first evidence for long-term transmission of CT’s toxic effect via extracellular vesicles was obtained in Chinese hamster ovary (CHO) cells. To follow the CT intracellular route towards exosome secretion, we used a novel strategy for generating metabolically-labeled fluorescent exosomes that can be counted by flow cytometry assay (FACS) and characterized. Our results clearly show the association of CT with exosomes, together with the heat shock protein 90 (HSP90) and Protein Disulfide Isomerase (PDI) molecules, proteins required for translocation of CTA across the ER membrane into the cytoplasm. Confocal microscopy showed direct internalization of CT containing fluorescent exo into CHO cells coupled with morphological changes in the recipient cells that are characteristic of CT action. Moreover, Me665 cells treated with CT-containing exosomes showed an increase in Adenosine 3’,5’-Cyclic Monophosphate (cAMP) level, reaching levels comparable to those seen in cells exposed directly to CT. Our results prompt the idea that CT can exploit an exosome-mediated cell communication pathway to extend its pathophysiological action beyond an initial host cell, into a multitude of cells. This finding could have implications for cholera disease pathogenesis and epidemiology.

## 1. Introduction

In the 21st century, cholera remains an epidemic or endemic disease in many parts of the world. It is caused by some serogroups of the bacterium *Vibrio cholerae* which colonize the small intestine and secrete the Cholera Toxin (CT) protein [1]. CT is made up of two major subunits, A and B [2], similar to other members of the AB5 family of toxins, and, once secreted by bacteria as a holotoxin, enters host cells by hijacking endogenous internalization and intracellular trafficking pathways, culminating in the induction of toxicity [3]. The A subunit (CTA) represents the enzymatic portion of the enterotoxin, and is composed of a globular A1 domain (CTA1), which possesses Adenosine 5’-diphosphate (ADP)-ribosylating activity, and the A2 domain (CTA2), that stabilizes the homo-pentameric B subunits (CTB) by noncovalent binding.

Internalization of CT depends on interaction of the CTB subunits of the toxin with GM1 gangliosides. GM1 gangliosides are typically concentrated in organized signaling centers such as lipid rafts and caveolae [4,5,6]. Localization of the cholera toxin within caveolae has triggered the idea that these sites may constitute clathrin independent carriers of the toxin. Although there is no evidence that CT enters cells specifically through the caveolae pathway, experiments have shown that GM1 and Caveolin-1 (Cav-1) expression levels are selective factors for the caveolae/raft-dependent endocytosis of cholera toxin [7].

Extracellular secretion gives rise to a variety of vesicles (EV), including those strictly derived from MVBs and properly defined as exosomes. Exosomes (exo) are vesicles of 30–150 nm diameter that are secreted by cells into their environment. They are generated by inward budding of endosomal membranes to form multivesicular bodies (MVBs). Fusion of MVBs with the plasma membrane typically releases multiple exosomes [8,9]. An increasing number of intracellular molecules has been reported to enter into exosomes and to be secreted in the extracellular space, suggesting a role for these vesicles as shuttles that deliver cargo molecules from one cell to another, and whose contents may be used for monitoring the metabolic state of the cell [10,11,12].

A few studies have examined the involvement of exo in toxin trafficking. The lethal factor (LF) of Anthrax toxin, a major *Bacillus anthracis* virulence factor, is translocated into the lumen of endosomal intraluminal vesicles (ILVs). It persists in them for days, and can be transmitted to neighboring cells via exosomes [13]. Trichosanthin (TCS), a plant toxin, is incorporated into intraluminal vesicles of the MVB, and is then secreted in association with exosomes upon fusion of the MVB with the plasma membrane [14]. In this paper, we show that that internalized CT molecules are sorted into MVBs, and are secreted as exosomes by Me665 and CHO cells. Furthermore, we show that CT contained in exosomes may be transferred to naïve recipient cells, and is able to induce morphological and functional changes typical of CT intoxication. To follow the transport of CT along the MVB/exosome route, we take advantage of a new methodology based on the fluorescent labeling of the phospholipid bilayer of exosomes that enabled us to trace and quantify exosome secretion [15].

## 2. Results

### 2.1. Extracellular Vesicles Isolated from CHO and Me665 Cells Upon CT Incubation Contain Cholera Toxin

**Figure 1:**
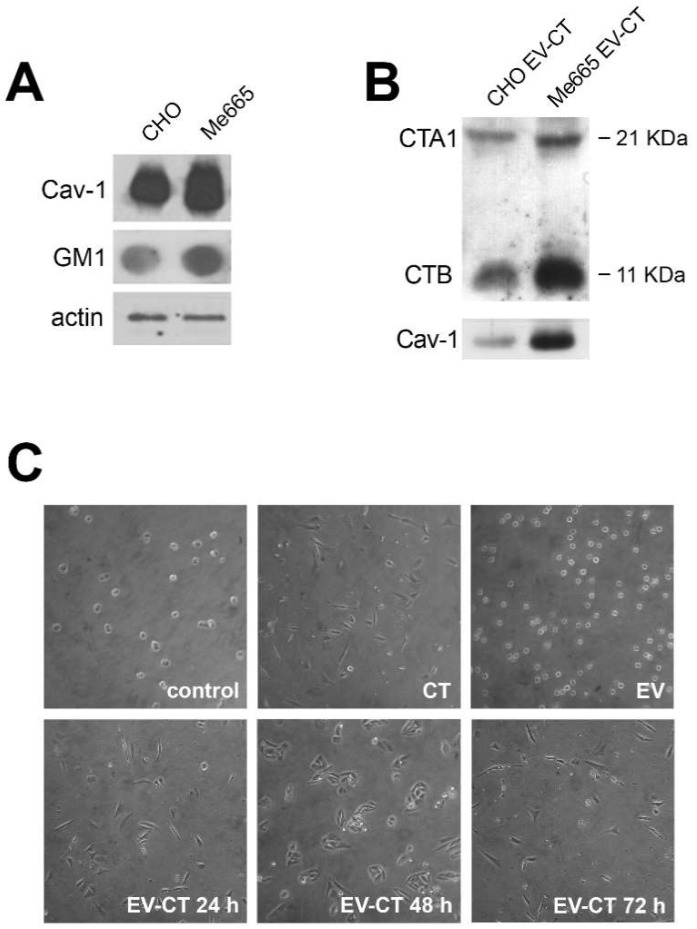
Expression of Cav-1 and GM1 in CHO and Me665 cells and morphological changes in CHO cells induced by CT-positive EVs (EV-CT) (**A**) Western blot analysis for Cav-1 and Dot blot analysis for GM1 of CHO and Me665 cell lines. For SDS-PAGE 30 µg of cell lysates were used and for Dot Blot 40 µg/dot. The presence of GM1 was assessed using horseradish peroxidase (HRP)-conjugated CTB. Actin is shown for normalization; (**B**) EVs collected from supernatants of CHO and Me665 cells treated with CT were run on a SDS-PAGE gel in reducing conditions Western blot analysis with a polyclonal antibody for Cav-1 and CT is shown; (**C**) Optical light microscope (Nikon, magnification ×10) analysis of CHO cells upon addition of extracellular vesicles from control or CT treated CHO cells. EV were collected at different times from cell-conditioned medium, and isolated by differential ultracentrifugation. After isolation, 5 µg of EV were incubated for 6 h with CHO cells. 12 nM CT is used as positive control. At the end of the incubation, cells were analyzed with a light microscope.

We previously reported that Cav-1, a structural component of caveolae formation, is highly expressed in human metastatic melanoma cell lines, and is retrieved in isolated fractions of extracellular vesicles (EV) [16]. Since caveolae are known locations for CTB binding to GM1 gangliosides, we hypothesized that Cav-1 and CT might share the endocytic pathway that leads to EV secretion. We first evaluated the relative levels of GM1 and Cav-1 in Me665 melanoma cells and CHO cells. Figure 1A shows that both cell types expressed these molecules, with higher levels in Me665 cells.

To determine whether CT was secreted in EVs, culture supernatants from CT treated Me665 and CHO cells (2 × 10^7^ cells for both cell lines) were subjected to differential centrifugation to isolate EVs, and vesicle pellets were analyzed by western blot for CT presence. Both CTA and CTB subunits were present in EV pellets from both cell types, and were slightly more abundant in Me665 cells (Figure 1B).

To determine if CT carried by EVs was capable of inducing morphological changes in cells that had not been directly exposed to CT, we used a well-established CHO cell morphological assay [17]. In this experiment, CHO cells were exposed to 12 nM CT for 2 h, washed, and incubated with fresh medium (without CT) for 24 h at 37 °C. After removal of the conditioned medium, fresh medium was added (twice), each time for an additional 24 h. EVs were purified from each of the conditioned media samples (EV-CT 24 h, EV-CT 48 h and EV-CT 72 h), and as a control, EVs were purified from replicate CHO cells treated in an identical manner, but without CT-exposure. To determine if the CT carried by EVs was capable of inducing biological effects in cells that had not been directly exposed to CT, we used a well-established morphological assay based on CHO cell morphology [17]. Freshly trypsinized CHO cells were treated for 6 h with 5 µg of EVs from the CT-exposed cells, or with 5 µg of EVs from control cells. As a positive control, replicates cells were directly exposed to 12 nM CT. Under normal conditions, most untreated cells remain round (Figure 1C) (even after overnight culture, not shown). In contrast, when directly exposed to 12 nM CT, the majority of cells became bipolar and elongated. In the case of cells exposed to EVs derived from CT treated cells, 60–80% became bipolar and elongated, comparable to cells exposed directly to 12 nM CT (Figure 1C). Notably, EVs secreted after 48 or 72 h from cells challenged with CT were also able to induce a morphological change, indicating that functionally active CT is present in the EVs.

### 2.2. Quantification and Characterization of Fluorescent Exosomes Containing CT Demonstrate the CTA Subunit Inclusion

**Figure 2:**
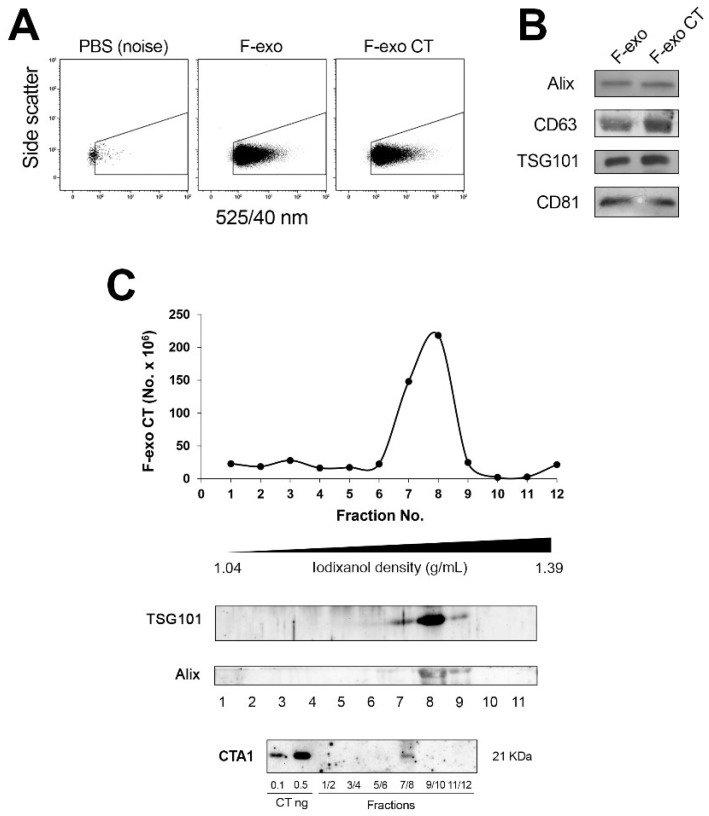
Characterization and distribution of F-exo CT purified from Me665 cells on an iodixanol gradient. (**A**) FACS analysis of F-exo and F-exo CT deriving from Me665 cells incubated with or without 12 nM CT. To design the F-exo region above instrument background noise only phosphate buffered saline (PBS) was acquired. Note that no events were registered in this region; (**B**) Western blot analysis of F-exo and F-exo CT probed with antibodies against exosome markers Alix, Tumor Susceptibility Gene (TSG)101, CD63 and CD81; (**C**) The F-exo CT sample was loaded at the bottom of an iodixanol discontinuous density gradient and subjected to ultracentrifugation for 18 h. The resulting fractions (1–12) with increasing density were analyzed for vesicles number by FACS and for the presence of exosome markers TSG101 and ALIX by Western blotting. The fluorescent peak displays a density ranging from 1.085 to 1.142. Fractions 1–2, 3–4, 5–6, 7–8, 9–10, 11–12 were pooled, trichloroacetic acid (TCA) precipitated and analyzed by western blot after running a Sodium dodecyl sulfate polyacrylamide gel electrophoresis (SDS-PAGE) in reducing condition for the presence of CTA subunit. For Western blot, an equal volume of each sample was analyzed.

To better define the role of exosomes as a carrier of CT, we applied a method we have developed based on the production of metabolically labeled fluorescent exo (F-exo), by using the fatty acid analogue BODIPY^TM^ FL C_16_ (4,4-Difluoro-5,7-Dimethyl-4-Bora-3a,4a-Diaza-s-Indacene-3-hexadecanoic Acid) [15]. Upon incubation with cells, BODIPY C16 enters the cellular lipid pathway and is transformed mostly into phospholipids that will become part of the F-exo lipid bilayer. Accordingly, we labeled Me665 cells for 5 h with BODIPY C16, followed (or not) by the addition of 12 nM CT for 2 h, and incubated for additional 24 h in fresh media. F-exo were purified by differential centrifugation from conditioned media, and F-exo were analyzed by FACS. As shown in Figure 2A, both F-exo and F-exo CT appear as discrete, countable fluorescent populations, comparable in size and fluorescence intensity.

Equal numbers of F-exo and F-exo CT were characterized by Western blot analysis for the presence of typical exo markers (Alix, TSG101, CD63 and CD81). Figure 2B shows that these markers are equally present in both F-exo and F-exo CT preparations, showing that CT does not alter the protein profile of the exosomes. To further characterize the F-exo population, we loaded F-exo CT onto a continuous iodixanol gradient to allow optimal separation of EV subtypes of different buoyant densities, and the gradient fractions were analyzed for vesicle number by FACS (Figure 2C). F-exo CT separated in a discrete peak of density range 1.085–1.142 g/mL, as measured by refractometry, in accordance with the previously reported density of exosomes [8]. Western blot analysis of gradient fractions showed that the exosomal markers TSG101 and Alix colocalized with the fluorescent peak (fractions 7–8), further indicating that the F-exo population corresponds to bona fide exosomes. The gradient fractions were also probed by western blot for the presence of CT with a monoclonal antibody against CTA. Results show that CTA1 was present in the pooled 7–8 fractions, corresponding to the F-exo fluorescent peak (Figure 2C). This shows the occurrence of the enzymatic moiety of CT in the exosomes.

### 2.3. Intracellular Distribution of CT in Me665 Cells

**Figure 3:**
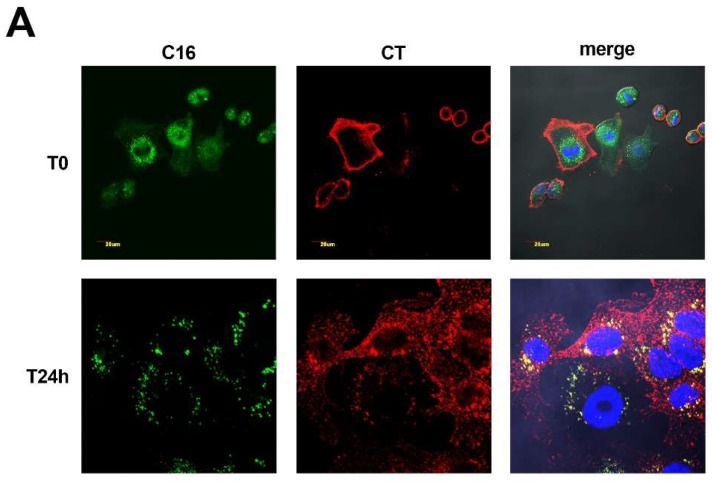

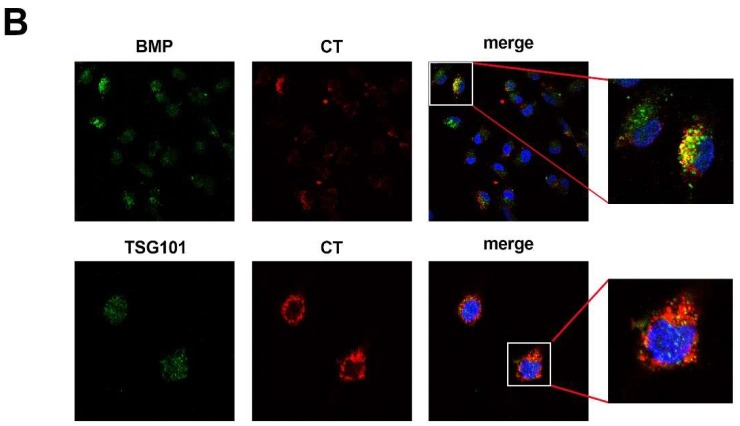
Intracellular distribution of CT in Me665 cells labeled with BODIPY C16. (**A**) Confocal microscopy analysis of Me665cells metabolically labeled with BODIPY C16, treated with 12 nM CT for 20 min on ice, and then incubated with CT-free medium for 24 h at 37 °C. Images were taken at T0 and T24 h after the removal of CT. BODIPY C16 is represented in green and CT in red; (**B**) Cells, immunolabeled for BMP or TSG101 (green) and for CT (red) show colocalization of both with CT, as evidenced in insets. Bar represents 20 µm. For all images DAPI staining was used for nuclear localization.

To visualize the intracellular trafficking of CT, we first pulsed Me665 cells with BODIPY C16 for 5 h to metabolically label membrane sub-compartments. Cells were then treated with 12 nM CT for 20 min on ice to allow CT binding to the plasma membrane. The cells were then washed (T0) and incubated with fresh medium for a further 24 h at 37 °C (T24). Following fixation, cells were analyzed by confocal microscopy. As shown in Figure 3A, at T0, CT clearly labeled only the plasma membrane and no co-localization with BODIPY C16-labeled internal membrane compartments was observed. Notably, lipid associated fluorescence was not present at the plasma membrane, further suggesting that F-exo do not derive from plasma membrane direct budding. At T24, CT staining was diffused in the cytoplasm, as well as in discrete spots, and co-localized with lipid-based fluorescence, suggesting the presence of CT in late endosomal/MVB compartments.

To further characterize the subcellular localization of CT, we performed confocal microscopy of CT-treated cells (T24) using antibodies against BMP, a lipid whose occurrence is specific to the late endosomal MVB/lysosomal membrane tracking pathway [18], and TSG101, a component of ESCRT that functions in the early stage of MVBs formation [19,20]. Figure 3B shows that CT co-localized with both BMP and TSG101 (inset), providing additional proof of the intersection of the CT trafficking pathway with the exosome biogenesis pathway.

### 2.4. CTA Active Subunit Is Retrieved in Association HSP90/PDI with Respect to F-exo Biogenesis

**Figure 4:**
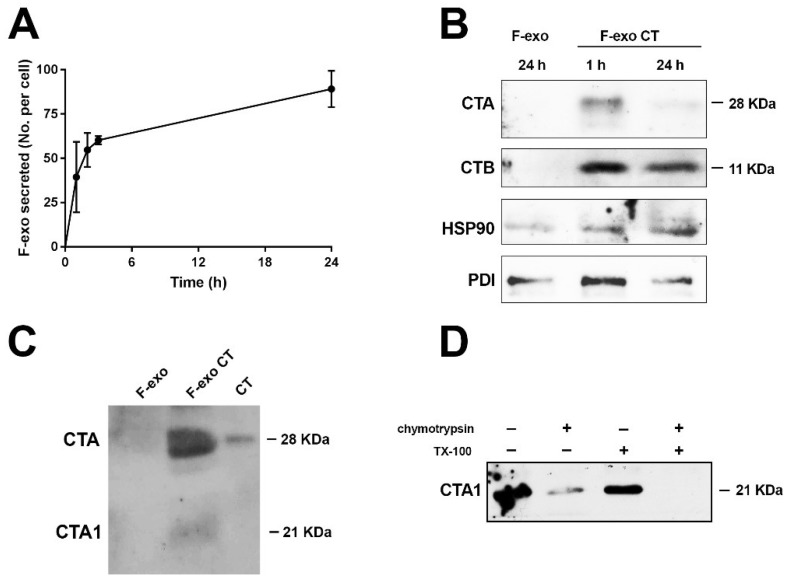
Exosome biogenesis and analysis of intra/extra localization of CT. (**A**) Me665 exosome biogenesis. Cells were pulsed with BODIPY C16 for 5 h for cell labeling, washed and complete medium was added. Cell conditioned medium was harvested at different time points for exosome recovery and quantification by FACS. Amount of exo secreted per cell is shown; (**B**) Western Blot analysis of F-exo CT recovered at 1 h and 24 h subtracted of the 1 h time point. The same number (3 × 10^7^) of F-exo or F-exo CT were run on a non reducing SDS-PAGE gel, and immunoblotted for CTA and CTB subunits, HSP90 and PDI; (**C**) Western blot analysis of F-exo and F-exo CT (6 × 10^7^) run in non-reducing conditions to show the presence of CTA1 and CTA1 + CTA2 subunits. The monoclonal antibody anti-CTA reveal the presence of both the 28 kDa and the 21 kDa subunits. 0.3 ng of CT were used as positive control; (**D**) 16 µg of exo CT were incubated with 1 µg of chymotrypsin, in the absence or presence of 0.2% Triton X-100. Samples were then loaded on SDS-PAGE gel in reducing conditions before western blot analysis using a monoclonal antibody anti-CTA.

With the aim of tracking F-exo CT biogenesis in Me665 cells, we pulsed cells with BODIPY C16 for 5 h to allow metabolic labeling of membrane compartments, including MVB before CT addition, for a further 2 h. Cells were then chased in fresh media for 1 h, before the media was removed and fresh media added for time intervals of up to 24 h. Exosome purification was performed on each of the conditioned media samples, to determine the kinetics of F-exo release into the extracellular medium (Figure 4A).

From as early as 1 h, cells secrete a discrete population of F-exo that can be FACS counted.

F-exo CT collected at 1 h and successively, after the addition of fresh medium, at 24 h (3 × 10^7^ exo for each time point), were run on SDS-PAGE and immunoblotted with antibodies against CTA and CTB subunits. Figure 4B shows that CTA was present in F-exo CT at both time points, and was more evident at 1 h than at 24 h, presumably because the reservoir of CT in the cells was slowly decreasing over time. In addition, the blots were probed for the presence of HSP90 and protein disulfide isomerase (PDI), proteins that are required for protein translocation of the unfolded CTA1 domain from the ER lumen to the cytosol [21]. Both HSP90 and PDI were present in the F-exo CT samples 1 h and 24 h, indicating that exo may constitute a membrane translocation system that facilitates the spread of CT toxicity to other cells, since PDI has the ability to dissociate the active CTA1 from the holotoxin. To see if the native 21 kDa CTA1 subunit was also present in the exosome vesicles, we loaded F-exo CT onto an SDS-PAGE gel in non-reducing conditions, prior to western blotting with an anti-CTA antibody. Figure 4C shows that both the 28 kDa CTA (CTA1 + CTA2) and 21 kDa CTA1 bands are visible. Finally, to assess the localization of CTA, we exposed F-exo CT to chymotrypsin digestion, with or without Triton X-100, assuming that the CT molecules enclosed within exo lumen would be protected from hydrolysis by the vesicle membrane (Figure 4D). Western blot analysis with an anti CTA monoclonal antibody showed that, in the absence of detergent, chymotrypsin degraded a substantial fraction of exo-associated CTA. However, complete digestion was observed in the presence of Triton X-100 and chymotrypsin. These results show that a portion of the CTA component of the exosomes is present within the lumen of vesicles.

### 2.5. Functional Activation of Adenylate Cyclase Following F-exo Direct Transfer to Cells

**Figure 5:**
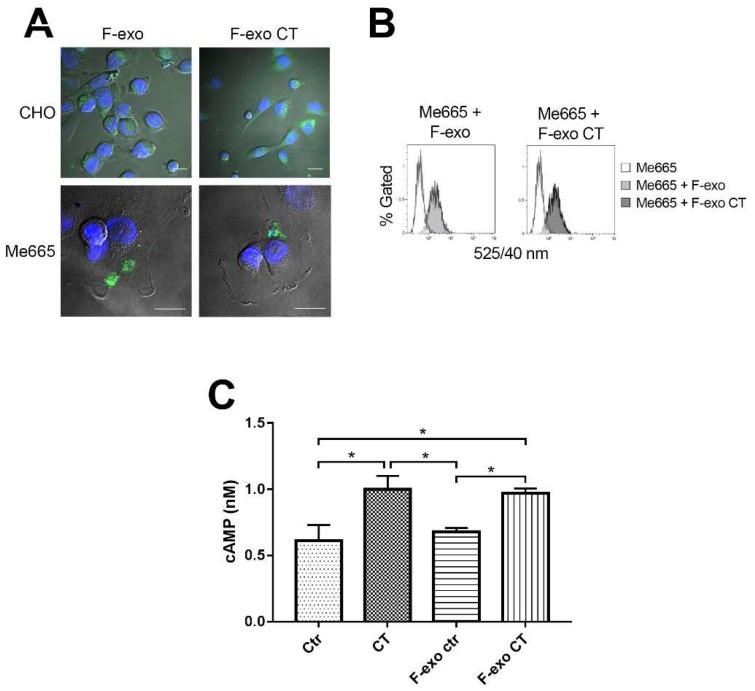
F-exo transfer to cells induces increase of cellular cAMP. (**A**) Confocal fluorescence microscopy images of F-exo and F-exo CT transfer on CHO and Me665 cells. 2 × 10^8^ fluorescent exosomes were incubated with 4 × 10^4^ CHO and Me665 cells for 4 h at 37 °C. Cells were then fixed and analysed; Scale bars represent 20 µm; (**B**) FACS analysis of F-exo and F-exo CT transfer on target cells. 4 × 10^4^ cells were incubated with 1.5 × 10^7^ F-exo or F-exo CT for 4 h at 37 °C. At the end of the incubation cells were FACS analysed. The increase in cell fluorescence demonstrate exo transfer to cells; (**C**) cAMP assay of Me665 cells treated with F-exo. 2 × 10^5^ cells were incubated with 5.7 × 10^7^ F-exo, F-exo CT or 0.2 ng/mL CT for 4 h at 37 °C. The graph shows the intracellular cAMP production of cells. * *p* < 0.05 Values are means ± S.D. (*n* = 3).

Exosomes play a central role in cell-to-cell communication by interaction with target cells. The influence of exosome based CT on CHO cell morphology indicates that the CT is activating intracellular adenylate kinase of target cells. To induce such a functional modification of the adenylate cyclase, giving rise to increased levels of cAMP [22], it is likely that exo containing CT must first be endocytosed by recipient cells. To test this hypothesis, we incubated CHO and Me665 cells with homologous F-exo containing CT, or with F-exo derived from control (non CT-exposed) cells. Internalization of F-exo was evaluated by confocal microscopy (Figure 5A).

When CHO cells were treated with control F-exo, uptake of the exosome-associated fluorescence was clearly visible, although no morphological changes were observed. In contrast, treatment with F-exo CT induced the characteristic elongated shape (Figure 5A upper panel). Internalization of F-exo fluorescence could also be observed in Me665 cells, both by confocal microscopy (Figure 5A lower panel) or by direct assessment of fluorescence transfer by FACS analysis (Figure 5B). Finally, to directly assess the activation of adenylate cyclase in Me665 cells, we treated cells with control F-exo, or F-exo CT for 4 h and measured cAMP levels by using a cAMP assay kit. Replicate cells were directly exposed to CT as a positive control, or were treated with neither CT nor exosomes. Exposure of cells to CT (0.2 ng/mL) or to F-exo CT increased cAMP levels to comparable extents, while control F-exo treatment did not affect cellular cAMP levels. By using fluorescence calibration beads, we could estimate that by incubating 285 exo per cell in the experiment in Figure 5C, only 35 exo per cell were taken up by recipient cells (means of 4 experiments, S.D. = 6.5). These data show that CT carried by exosomes is biologically active in target cells.

## 3. Discussion

Cholera toxin cell entry and intracellular trafficking has been widely studied. There is a common consensus that GM1-based binding of CT is followed by retrograde vesicular trafficking from the plasma membrane through early endosomes, the Trans-Golgi Network, and the endoplasmic reticulum (ER), before the active CTA1 subunit reaches the cytosol and its final specific substrate [23]. This latter step appears to require interactions with host cell accessory proteins, such as the chaperone Hsp90 and the protein-folding helper enzyme PDI, which allow membrane translocation of the unfolded CTA1 domain [4,24,25]. Once in the cytosol, CTA1 recovers the active conformation required for ADP-ribosylation of its target, the α subunit of the heterotrimeric G protein (Gsα) [26,27] activates adenylate cyclase, giving rise to increased levels of cellular cyclic AMP (cAMP) [28]. It is well known that CTB binding and cross-linking of five GM1 molecules serve to promote the function of lipid rafts in toxin trafficking; in fact, lowering the expression of GM1 or cholesterol can prevent CT endocytosis [29,30,31]. However, the specific intracellular trafficking pathway of GM1 is not well understood. Despite the fact that many aspects of the CT-GM1 retrograde trafficking pathway from PM to ER have been elucidated, when three major pathways of CT entry (i.e., caveolin, clathrin, or Arf6 dependent) are inhibited, no significant reduction in toxicity is observed, and therefore, one or more as yet unknown pathways have been hypothesized to contribute [32]. A retracing study of CT intoxicating retrograde pathways carried out using subsets of GM1 (specified by saturated or unsaturated fluorescent-labeled ceramide chains) showed that CT bound to saturated GM1, in contrast to that bound to the unsaturated complex forms, is directed towards late endosome compartments (including MVBs that contain exosomes) rather than towards the classical Golgi/ER pathway [33]. Accordingly, it has been reported that GM1 gangliosides localize in the membrane of exosomes [34]. Notably, previous studies showed that caveolae and lipid raft associated proteins and lipids can take part in biogenesis and secretion of cell-derived exosomes [16,35,36].

In this study, we reported for the first time that CT can be propagated via EVs in a biologically active form, and that CT persists in cells long after treatment with the toxin. Accordingly, EVs collected from cells up to 72 h after their exposure to CT, still contain biologically active CT. Cells release different types of EVs, not all of endo/lysosomal origin, such as ectosomes that directly bud from the plasma membrane [18]. To distinguish among different types of vesicles, we applied a novel methodology that was developed in our lab [15] for the metabolic labeling of exosomes. BODIPY C16, a fluorescent phospholipid precursor, is efficiently incorporated into cells, and once metabolized in the ER, takes the endosomal/MVB route to become an integral part of the exosome membrane. Notably, as shown in Figure 3A, lipid associated fluorescence seemed completely absent from the plasma membrane, whereby we could exclude its involvement in fluorescent ectosomes generation.

Fluorescent exo secreted in the extracellular medium can be purified and readily counted. It has also to be noted that the final purified vesicular pellet contains much less vesicles than those present in the initial medium. In fact, our labeling technique allowed us to determine that a considerable amount of fluorescent exosomes remain suspended or associated with different kind of debris and serum macromolecular components in the supernatant and in the low speed pellets. We show here that CTA is associated with a discrete population of fluorescent exo that express typical exo markers, indicating that CT is a bona fide component of exosomes (Figure 2). Furthermore, this methodology allowed us to highlight intracellular membrane compartments that intersect with the trafficking pathway of internalized CT (Figure 3A). Specifically, colocalization of CT with MVB-specific markers was observed in the endolysosomal compartment (Figure 3B). This suggests that exosomes, in addition of being a means of transport, may represent a kind of CT-repository within the MVBs, allowing CT to be protected from intracellular degradation, and allowing for long-term activity. Despite the fact that in vitro uptake of exosomes is a complex mechanism which has not yet been clarified, we also demonstrate that there is a strict relation between the cellular uptake of CT containing exo and functional alterations in recipient cells highlighted in CHO cells by the morphological changes and in Me665 cells by direct increase of cellular cAMP levels. A final consideration is that in a physiological setting, there may be many other factors that can influence the efficiency of cellular uptake, such as time of exposure, or more importantly, the vicinity between intoxicated cell secreting exosomes and recipient cells.

F-exo biogenesis studies allowed us to monitor CT secretion over time, highlighting an early release of F-exo containing CT (at 1 h). At later times (24 h), CT was still present in F-exo, albeit in lower amounts, showing that the bulk of CT associated with exo is released soon after toxin internalization. Interestingly, the host cell chaperone HSP90, an established exosomal marker [37], and protein-helper enzyme PDI were both clearly present in the exosomes, together with the CT subunits (Figure 4B). Furthermore, CTA was present in exo both in the active reduced form, CTA1 (21 kDa), or as the inactive disulfide linked CTA1 and CTA2 (28 kDa) form. Chymotrypsin digestion of F-exo containing CT revealed that the CTA subunit is distributed both outside and inside of exosome membranes. Based on our results, an appealing speculation is that CTA associated with exo may derive from two distinct intracellular routes: upon cell entry, some CT molecules may follow the long chain saturated GM1 pathway [33], presumably along with Cav-1, leading the CT holotoxin direct to the late endosomal compartment/MVB before being secreted; on the other hand, CTA1 in exosomes may originate from the canonical ER retrograde pathway, where, by the action of HSP90 and PDI, CTA1 is translocated to the cytosol and taken up during exo formation by intraluminal vesicles.

In conclusion, starting from the more general hypothesis which suggests that exosomes are vehicles that can influence cell homeostasis, we found that CT could exploit the exosome biogenesis pathway to spread its action from a single cell to an entire cell population. In light of our results, we may hypothesize that *V. cholerae* fast dynamic mechanism of intestinal infection relies on the effect of the cell-to-cell transmission of CT containing exo. In fact, in normal healthy adults, primary symptoms of cholera disease appear from half a day to five days after ingestion of at least 100 million bacteria, of which only few thousands survive the harsh acidic conditions of the stomach. Afterwards, only a small handful of bacteria penetrate the thick mucus wall to reach the *V. cholerae* natural setting, the small intestine, where they take hold and release CT [38]. We reason that the rapidity by which infection propagates among lining intestinal epithelia may be caused by the horizontal transfer of exosomal payloads that include active CTA subunits. This latter readily interacts with the host cell mechanisms to pump chloride ions into the small intestine, which can pull up to six liters of water per day through the intestinal cells through osmosis, eventually creating a thriving environment for bacteria exponential growth. Thus, our study suggests potentially important roles for exosomes in cholera pathogenesis, and in the dynamics of this infectious disease, given that these vesicles seem to preserve and protect the toxin, and facilitate its transfer from cell to cell.

## 4. Materials and Methods

### 4.1. Cell Culture

The Human melanoma cell line, Me665/1 (Me665), stabilized from surgical specimen obtained from metastatic lesions at Istituto Nazionale Tumori (Milan, Italy) and CHO (Chinese hamster ovary, American Type Culture Collection, Rockville, MD, USA) cell line commercially available, were grown in Dulbecco’s modified Eagle’s medium (DMEM) (EuroClone, S.p.A. Milan, Italy) supplemented with 10% fetal bovine serum (FBS) (Biological Industries, Kibbutz Beit Haemek, Israel), 5 mM l-glutamine, penicillin (100 units/mL), and streptomycin (100 μg/mL) (complete media, EuroClone S.p.A. Milan, Italy) at 37 °C in a humidified 5% CO_2_ atmosphere.

### 4.2. Isolation of EVs from CHO and Me665 Cell Culture Supernatants

For isolation of EVs, subconfluent monolayers of CHO and Me665 cells in exponential growth were incubated in DMEM supplemented with 0.3% FBS with or without 12 nM CT, and purified from culture filtrates of *Vibrio Cholerae* 569 B, serotype Inaba, as described by [39]. After 2 h of CT treatment, control and CT treated cells were washed in PBS and further cultured in fresh DMEM medium for 24 h before collection of medium for EVs isolation. EVs were isolated from the culture supernatants of treated and untreated cells by sequential centrifugations, as previously described [15,40], with some modifications. Briefly, culture supernatant was centrifuged at 2000× *g* for 20 min at 4 °C to pellet cells debris. Supernatants were transferred to new tubes and centrifuged in a SW41Ti rotor (Beckman, Coulter, Milan, Italy) at 10,000× *g* for 20 min at 4 °C, and finally ultracentrifuged at 100,000× *g* for 3 h. Pellets were washed in PBS and ultracentrifuged at the same speed for 3 h to further remove any residual CT still present in the exo preparation, since, along with vesicles, some free intact CT is also recycled to the extracellular milieu, and can be found in the supernatant after ultracentrifugation.

### 4.3. Morphological Analysis of CHO Cells after CT, EV and EV-CT Treatment

Exponentially growing CHO cells were harvested, transferred to 12-well plates (2 × 10^4^ cells/well), and DMEM supplemented with 0.3% FBS. Cells were incubated with or without 12 nM CT, or with EV and EV-CT (prepared as described above). After 6 h incubation, cells were inspected microscopically to analyze the morphological changes.

### 4.4. Dot Blot and Western Blot Analysis

Cell pellets were lysed in 20 mM Tris, pH 7.4, 150 mM NaCl, 1% Triton X-100, 2 mM EDTA with a Protease Inhibitor Cocktail (Roche Applied Sciences, Mannheim, Germany) for 20 min on ice, and then centrifuged at 2000× *g* for 10 min. The pellet was discarded and supernatant was kept for further analysis. Protein concentration was measured using the BCA assay (Thermo Fisher Scientific, Waltham, MA, USA). The presence of GM1 ganglioside in cell lysates was assessed by the dot blot assay using horseradish peroxidase (HRP)-conjugated CTB subunit, and revealed with an ECL detection kit (Pierce^TM^ ECL Western Blotting Substrate, Thermo Fisher Scientific). Cell lysates (40 µg) were boiled and spotted onto a nitrocellulose membrane blocked using 5% Blotting Grade non-fat dry milk in a TBS-Tween (TBST) buffer (10 mM Tris-HCl (pH 8.0), 150 mM NaCl, 0.1% Tween 20) for 45 min at room temperature (RT), followed by incubation with HRP-CTB dissolved in TBST buffer (1:400) for 1 h at RT. The reactivity was detected using an ECL detection kit (Pierce). For Western Blot analysis, exosome preparations were lysed in Laemmli sample buffer, boiled, and loaded onto 10% or 14% SDS-PAGE gels under reducing or non-reducing conditions. Proteins were blotted onto a nitrocellulose membrane, and blocked using 5% Blotting Grade non-fat dry milk in TBS-Tween (TBST) buffer (10 mM Tris-HCl (pH 8.0), 150 mM NaCl, 0.1% Tween 20) for 1 h at RT followed by incubation with primary antibodies. The following primary antibodies were used: mouse anti-Cholera Toxin A (Clone 2H9) (Immunology Consultants Laboratory, Inc., Portland, OR, USA) 1:500 and rabbit polyclonal Anti-Cholera toxin (Sigma Immuno Chemicals, St. Louis, MO, USA) 1:1000; rabbit anti-Caveolin-1 (N-20) (Santa Cruz Biotechnology, Dallas, TX, USA) 1:1000; rabbit anti-Protein Disulfide Isomerase Polyclonal Antibody (Stressgen Biotechnologies Corporation, San Diego, CA, USA) 1:1000; rabbit anti-HSP90 α/β (H-114) (Santa Cruz Biotechnology) 1:1000 dissolved in TBS-Tween (TBST) buffer for 1 h at RT; mouse anti-Alix (3A9) (Thermo Scientific) 1:1000, rabbit anti-CD63 (SBI) 1:1000, mouse anti-CD81 1:1000, mouse anti-TSG101 (GeneTex, Inc., Irvine, CA, USA) 1:1000 dissolved in 0.25% Blotting Grade non-fat dry milk in TBST overnight at 4 °C. After washing with TBST, filters were incubated with appropriate horseradish peroxidase-conjugated secondary antibodies (Bio-Rad, Hercules, CA, USA) for 1 h at RT, and immunoreactivity was revealed by using an ECL detection kit (Pierce).

### 4.5. Generation and Quantification of F-exo

F-exo were purified and quantified as previously described [15]. Briefly, melanoma cells Me665 (8 × 10^5^) were incubated with 7 µM BODIPY FL C16 (4,4-difluoro-5,7-dimethyl-4-bora-3a,4a-diaza-s-indacene-3-hexadecanoic acid) (BODIPY C16) (Life Technologies, Carlsbad, CA, USA) for 5 h at 37 °C in DMEM supplemented with 0.3% FBS. Excess probe was removed by washing cells twice with PBS. Subsequently, cells were incubated with or without 12 nM CT in DMEM supplemented with 0.3% FBS for 2 h at 37 °C, and washed twice with PBS to remove excess CT and further incubated for different times in complete media. Conditioned medium was centrifuged at 2000× *g* for 20 min to remove cells and cell debris. The pellet was discarded and the supernatant centrifuged at 10,000× *g* (10 K) to remove microvesicles. F-exo and F-exo CT were isolated by ultracentrifugation of the 10 K supernatant at 100,000× *g* (100 K) for 3 h, and the resulting pellet washed in PBS for 3 h at 100,000× *g*. All ultracentrifugation steps were performed at 4 °C using a SW41 Ti rotor (Beckman Coulter, Brea, CA, USA). The final pellet was resuspended in PBS and quantified by FACS (FC Gallios Flow Cytometer and Kaluza Software-Beckman Coulter). To set the instrument, fluorescent beads (525/540 nm FL1) ranging in size from 0.1 to 0.5 µm were analyzed. Flow count fluorospheres, used to determine exosome number, were resuspended in PBS with F-exo and F-exo CT, and the instrument was set to fix the stopping gate on 2000 flow count fluorospheres. For further details, refer to [15]. For F-exo biogenesis studies, F-exo CT were collected at different time points (1–24 h) after 2 h of CT incubation.

### 4.6. Iodixanol/OptiPrep^TM^ Gradient Separation

A discontinuous iodixanol gradient was used for further exosome purification, as described by several authors [41]. Solutions of 10%, 30% and 40% iodixanol were made up by mixing appropriate amounts of homogenization buffer (HM solution: 0.25 M sucrose, 1 mM EDTA, 10 mM Tris-HCL pH = 7.4) and an iodixanol working solution. This working solution was prepared by combining a working solution buffer (0.25 M sucrose, 6 mM EDTA, 60 mM Tris-HCL pH = 7.4) and a stock solution of OptiPrep^TM^ (60% *w*/*v* aqueous iodixanol solution, Sigma-Aldrich, Saint Louis, MS, USA). The gradient was formed by layering 1 mL of 60% with 0.260 mL sample, 0.5 mL 40%, 0.5 mL 30% and 1.8 mL of 10% solutions on top in a 4.5 mL open top polyallomer tube (Beckman Coulter, Brea, CA, USA). The gradient was then centrifuged for 18 h at 192,000× *g* at 4 °C (SW60 Ti rotor Beckman Coulter). Gradient fractions of 350 µL were collected from the top of the gradient and analyzed by FACS for exosome count. Fractions were loaded on a SDS-PAGE gel for Western blotting analysis using antibodies directed against exosome markers TSG101 and Alix. For CT detection, gradient fractions were TCA precipitated. The refractive index of each fraction was assessed with a refractometer (Carl Zeiss, Oberkochen, Germany), and the relative density was calculated using the linear relationship between refractive index (η) and the density (ρ) ρ = Aη − B.

### 4.7. Chymotrypsin Treatment of exo

To analyze the intra/extra localization of CT, exo CT (16 µg) were incubated with or without 1 µg of chymotrypsin, in the absence or presence of 0.2% Triton X-100, at 37 °C for 3 h at pH 7.8. Digestion was stopped on ice with 5 µM phenylmethanesulfonyl fluoride (PMSF). Samples were then mixed with Laemmli buffer, heated at 95 °C for 5 min, and analyzed by SDS-PAGE and western blotting.

### 4.8. cAMP Assay

Freshly trypsinized cells were transferred to 6-well plates (2 × 10^5^ cells/well) and incubated overnight at 37 °C in DMEM with 10% FBS to allow cell attachment. Plates were then washed with serum-free DMEM, and 1 mL of 1% FBS DMEM containing 0.5 mM 1-methyl-3-isobutylxantine (IBMX, Sigma-Aldrich, Saint Louis, MO, USA) was added for 30 min. As control, IBMX treated cells were used. After incubation, 0.2 ng/mL of CT or a specific number (5.7 × 10^7^) of F-exo and F-exo CT were added and plates were incubated at 37 °C for 4 h. Cells were washed in ice-cold PBS, and cAMP assay was performed, according to manufacturer’s instructions (Cyclic AMP XP Assay kit, Cell Signaling Technology, Danvers, MA, USA).

### 4.9. Confocal Microscopy

To analyze the intracellular localization of CT, Me665cells (4 × 10^4^) were cultured on 24-well plates containing coverslips until 60–70% of confluency. Cells were incubated at 37 °C with or without 7 µM BODIPY C16 for 4 h. 12 nM CT was added for 20 min on ice (T0), and cells were then incubated at 37 °C for 24 h (T24). Cells were fixed with 3 % paraformaldehyde (PFA) for 20 min at RT and permeabilized with 0.05% saponin. The primary antibodies used were: rabbit polyclonal anti-Cholera toxin (Sigma-Aldrich), mouse anti-TSG101 (GeneTex, Hsinchu, Taiwan), and mouse anti-LBPA/BMP (6C4) (Echelon Corporation, San Jose, CA, USA). AlexaFluor647-conjugated and AlexaFluor488-conjugated goat anti mouse or anti rabbit (Life Technologies) was used as secondary antibody. Coverslips were mounted on the microscope slide with Vectashield antifade mounting medium containing DAPI (Vector Laboratories, Burlingame, CA, USA). Images were taken by a FV1000 confocal microscope (Olympus, Tokyo, Japan), using a (Olympus) planapo objective 60x oil A.N. 1.42. Excitation light was obtained by a Laser Dapi 408 nm for DAPI, an Argon Ion Laser (488 nm) for FITC (Alexa 488), and a Red Diode Laser (638 nm) for Alexa 647. DAPI emission was recorded from 415 to 485 nm, FITC emission was recorded from 495 to 550 nm, and Alexa 647 from 634 to 750 nm. Images recorded have an optical thickness of 0.3 mm.

### 4.10. F-exo and F-exo CT Transfer

To evaluate the transfer on target cells, F-exo were incubated with cells in a 24-well plate in duplicate in 1 mL DMEM with 0.1% FBS and kept for 4 h at 37 °C in CO_2_ incubator. The medium was then removed, and cells PBS washed, detached and subjected to FACS analysis for cell fluorescence, together with exo and a standard curve setup with Quantum™ FITC-5 MESF (Molecules of Equivalent Soluble Fluorophores) (Bangs Laboratories, Inc., Fishers, IN, USA). The amount of transferred exo was estimated, as previously described [15]. Briefly, we transformed fluorescence data (arithmetic mean) of exo using the QuickCal analysis template provided with each Quantum™ MESF lot to determine MESF per Exo and cells. To transform MESF associated to cells in number of Exo transferred, we used the formula: transferred Exo number = (cell fluorescence (MESF) − autofluorescence (MESF))/MESF associated to a single Exo. For confocal microscopy, 4 × 10^4^ cells were seeded on a sterilized coverslip and incubated with 2 × 10^8^ F-exo, or F-exo CT for 4 h at 37 °C in DMEM with 0.1% FBS. At the end of incubation, the medium was removed, and the cells were washed with PBS and fixed with 3% PFA for analysis.
